# Imbalance of Controlled Death in Peripheral Blood Lymphocytes in Crohn’s Disease and Ulcerative Colitis

**DOI:** 10.3390/medicina55060231

**Published:** 2019-05-31

**Authors:** Ewa Dudzińska, Kinga Szymona, Paulina Gil-Kulik, Piotr Chomik, Małgorzata Świstowska, Magdalena Gryzińska, Janusz Kocki

**Affiliations:** 1 Chair of Public Health, Faculty of Health Sciences, Medical University, 20-059 Lublin, Poland; 2 Department of Psychiatry, Psychotherapy and Early Intervention, Medical University, 20-059 Lublin, Poland; kszymona@ahe.lodz.pl; 3 Department of Clinical Genetics, Medical University, 20-059 Lublin, Poland; paulina.gil-kulik@umlub.pl (P.G.-K.); piotr.chomik@umlub.pl (P.C.); malgorzata.swistowska@umlub.pl (M.Ś.); janusz.kocki@umlub.pl (J.K.); 4 Institute of Biological Basis of Animal Production, Sub-Department of General and Molecular Genetics, University of Life Sciences in Lublin, 20-033 Lublin, Poland; magdalena.gryzinska@up.lublin.pl

**Keywords:** Crohn’s disease, ulcerative colitis, apoptosis

## Abstract

*Background and objectives*: Inflammatory bowel disease (IBD) mainly includes Crohn’s disease (CD) and ulcerative colitis (UC). Both conditions are associated with an exacerbated intestinal immune response to harmless stimuli, leading to upregulation of pro-inflammatory mediators. *Materials and Methods*: The subjects of the study were 55 patients with IBD. The control group consisted of 35 healthy subjects. The researched material consisted of peripheral blood lymphocytes collected from the subjects. Expression of the genes *BAX*, *BCL2*, *CASP3* and *CASP9* was assessed at the mRNA level in the peripheral blood lymphocytes of patients with ulcerative colitis and Crohn’s disease relative to the healthy subjects. The expression of the genes was determined by rtPCR using TaqMan probes specific for these genes. *Results*: The group of patients diagnosed with CD had statistically significantly higher expression of the genes *BAX* (*p* = 0.012), *BCL2* (*p* = 0.022), *CASP3* (*p* = 0.003) and *CASP9* (*p* = 0.029) than healthy subjects. Expression of *BAX*, *BCL2*, *CASP3* and *CASP9* in UC patients in the active phase of the disease was significantly lower than in patients in remission: *BAX* (*p* = 0.001), *BCL2* (*p* = 0.038) and *CASP9* (*p* = 0.007). In patients with UC, the BAX/BCL2 ratio was significantly correlated (r = 0.473) with the duration of the disease. In the group of CD patients treated biologically, a significantly lower BAX/BCL2 ratio was demonstrated than in patients that were not biologically treated. *Conclusions*: Our research has shown a simultaneous increase in the expression of the anti-apoptotic *BCL2* gene and the proapoptotic *BAX* gene, which suggests the dysregulation of apoptosis mechanisms in IBD. Significantly higher expression of *BAX* and *BCL2* in UC patients in remission as compared to CD may suggest differences in these diseases in terms of prognosis and treatment. Our results may suggest that an underlying imbalance in factors controlling apoptosis in peripheral blood lymphocytes may be the response of the immune system to inflammation of the intestinal mucosa. Modulation of apoptosis may become an important therapeutic mechanism in IBD.

## 1. Introduction

The term ‘inflammatory bowel disease’ (IBD) comprises Crohn’s disease (CD) and ulcerative colitis (UC). Both are chronic, relapsing gastrointestinal disorders associated with an exaggerated intestinal immune response to innocuous stimuli, resulting in upregulation of pro-inflammatory mediators [1].

Excessive activation of lymphocytes may be a factor in the pathogenesis of IBD [2].

Peripheral white blood cells of UC patients have been shown to have a cytotoxic effect on human foetal colon cells in vitro. Studies have shown thattheexpression of early activation markers on the intestinal lymphocytes and peripheral blood lymphocytes is increased and similar in Crohn’s patients. Rapid accumulation of phagocytes in the intestine of IBD patients has been observed as wellandwas correlated with the disease activity. All peripheral immune cells migrated and recirculated from the intestine to the blood and vice versa, which is believed to be an important factor in IBD pathogenesis [3].

Naive T helper (Th) cells are attracted to the site of inflammation, where factors released byantigen-presenting cells and other stromal elements mediate their differentiation. Activated T cells are involved in the barrier disruption and tissue damage that characterize IBD [4].

There are several medical treatments associated with BCL2-mediated apoptosis, as a BCL-2 protein imbalance can lead to unsuccessful therapeutic responses [2].

IBD is mainly treated with sulfasalazine, aminosalicylates, immunomodulators, steroids, and biopharmaceuticals. Treatments that regulate proliferation and contraction of lymphocytes are directly associated with BCL2-mediated apoptosis. Sulfasalazine is a pro-apoptotic drug, as demonstrated in peripheral blood lymphocytes and T cells of thelamina propria isolated from inflammatory lesions in Crohn’s patients. Sulfasalazine-induced apoptosis is linked to a decrease in anti-apoptotic BCL-XL and BCL2 [2].

Intracellular apoptosis involves both extrinsic and intrinsic (metabolic) pathways. The intrinsic pathway is controlled by BCL2-family proteins [5].

The mitochondrial-mediated apoptosis pathway is regulated by pro-apoptotic proteins BAX, BAD and BAK and byanti-apoptotic proteins BCL2, BCL-Xl and MCl-1. There have been reports indicating that an increased BAX/BCL2 ratio upregulates caspase-3, which in turn increases the level of apoptosis [6].

In this study, we evaluate the expression of the *BAX*, *BCL2*, *CASP3* and *CASP9* genes at the mRNA level in the peripheral blood lymphocytes of patients with ulcerative colitis and Crohn’s disease during the disease process. Weanalysedgenes in patients whose disease was active and patients in remission, as well as in those undergoing biological therapy.

## 2. Material and Methods

The subjects of the study were IBD55 patients diagnosed at the Gastroenterology Ward and Gastroenterology Clinic of the Cardinal Stefan Wyszyński Regional Specialist Hospital in Lublin. The control group (CTR), consisted of 35 healthy subjects (9 males and 26 females, aged 19–67, mean = 40 years).

Among the 27 CD patients, 25 were being treated with aminosalicylates (Sulfasalazine, Salofalk, Pentasa, or Asamax), 17 with azathioprine (Imuran or Azathioprine VIS), and 5with corticosteroids (Encorton), while 10 patients were receiving biological treatment (eight patients with Humira and two with Inflectra).

Of the 28 UC patients, 25 were being treated with aminosalycilates (Asamax, Pentasa, Salofalk), 11 with azathioprine (Imuran), and 4with corticosteroids (Encorton).

The severity of Crohn’s disease was assessed according to the Crohn’s disease activity indicator CDAI (remission <150 score, severe disease >450 score). The Mayo score (0—normal, 3—severe disease) was used to assess the severity of ulcerative colitis (Table 1).

A 4.9 mL volume of peripheral blood was collected from the IBD patients and the control group into tubes with EDTA as an anticoagulant. Within 2hours of collection, blood lymphocytes were isolated using Gradisol L and PBS density gradient centrifugation (Biomed, Lublin Poland). Then complete cellular RNA was isolated according to Chomczynski and Sacchi [7]. The purity and concentration of the RNA were measured, followed by a reverse transcription reaction.

The isolated RNA was used to conduct a cDNA synthesis reaction using reagents provided in High-Capacity cDNA Transcription Kits (Applied Biosystems, Foster City, CA, USA) with an RNaseinhibitor. Synthesis was carried out using 1 µg of RNA dissolved in 10 µL of ultrapure water. The reactions were performed in a 20 µL volume according to the manufacturer’s protocol, in the following conditions: 10 min at 25 °C, 120 min at 37 °C and 5min at 85 °C (Veriti Dx Thermal Cycler, Applied Biosystems).

Relative expression of the genes *BAX*, *BCL2*, *CASP3* and *CASP*9 was evaluated by qPCR using TaqMan-specific probes. Expression of the *GAPDH* gene was used as an endogenous control. The reaction was carried out in StepOne Plus (Applied Biosystems) in 96-well plates in a reaction volume of 25 μL per well, including 1 μL cDNA; 12.5 μL Gene Expression Master Mix buffer (Applied Biosystems); 10.25 μL ultrapure water; 1.25 μL specific probe for the test gene (Hs00180269_m1 for *BAX*, Hs00608023_m1 for *BCL2*, Hs00234387_m1 for *CASP3*, Hs00154261_m1 for for *CASP9* and Hs99999905_m1 for *GAPDH*, Applied Biosystems). The qPCR reaction was performed under the following conditions: 10 min pre-denaturation at 95°C followed by 40 cycles: 15 s at 95°C and 60 s at 60°C. After the reaction was complete, the results were analysed using ExpressionSuite V. 1.0.3 (Life Technologies, Carlsbad, CA, USA). Expression (RQ) of the *BAX*, *BCL2*, *CASP3* and *CASP9* genes in the control samples was tested using RQ = 2−ΔΔCt [8]. Statistical analysis of the results was performed using the Student’s t-test and Pearson’s correlation coefficients.

The study was conducted according to a protocol approved by the Local Bioethics Committee (approval: KE-0254/179/2016, approved on 23.06.2016).

## 3. Results

**Figure 1:**
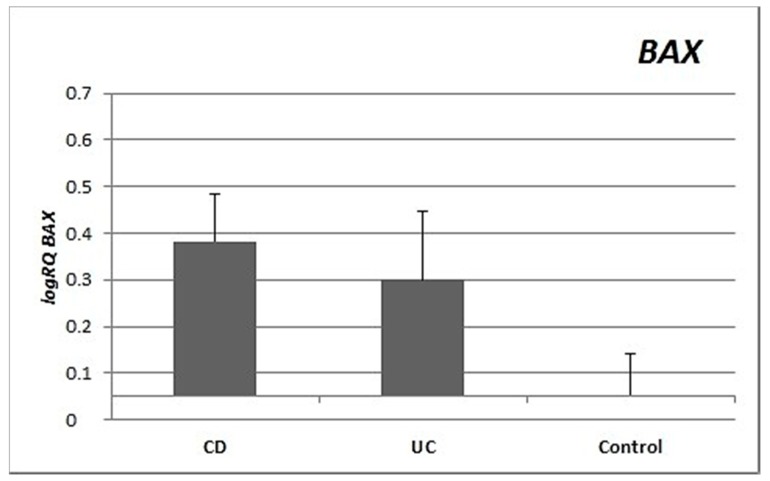
Mean expression (RQ) of the *BAX* gene at the transcript level in peripheral blood lymphocytes of patients with ulcerative colitis (UC) and Crohn’s disease (CD) relative to the mean expression of the gene in the peripheral blood lymphocytes of the control group (calibrator). The result shows the average relative expression in the group (RQ ± SEM). * *p* < 0.05 (Student’s t-test).

Expression of the *BAX* gene in the peripheral blood lymphocytes was twice as high and statistically significantly (*p* = 0.012) higher in CD patients compared to controls, and nearly twice as high in UC patients as in the controls (*p* = 0.32). There were no significant differences in *BAX* gene expression between lymphocytes from patients with CD vs UC (*p* = 0.332) (Figure 1).

**Figure 2:**
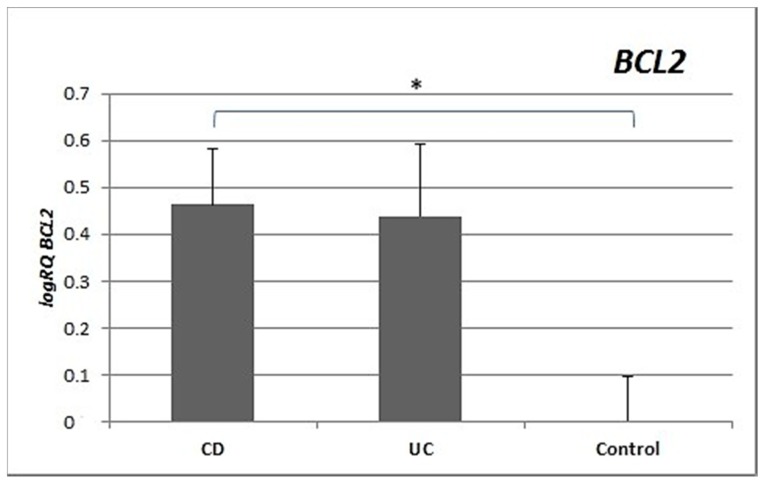
Mean expression (RQ) of the *BCL2* gene at the transcript level in peripheral blood lymphocytes of patients with UC and CD relative to the mean expression of the gene in the peripheral blood lymphocytes of the control group (calibrator). The result shows the average relative expression in the group (RQ ± SEM). * *p* < 0.05 (Student’s t-test).

Expression of the *BCL2* gene in the peripheral blood lymphocytes of patients with Crohn’s disease was significantly and nearly three times higher than in controls (*p* = 0.022). There were no significant differences in the expression of *BCL2* between the group with UC and controls (*p* = 0.249) or between UC patients and CD patients (*p* = 0.336) (Figure 2).

**Figure 3:**
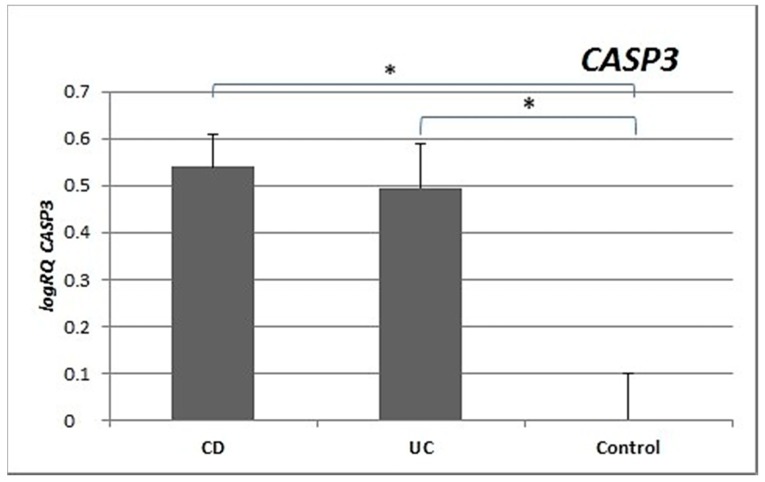
Mean expression (RQ) of the *CASP3* gene at the transcript level in peripheral blood lymphocytes of patients with UC and CD relative to the mean expression of the gene in the peripheral blood lymphocytes of the control group (calibrator). The result shows the average relative expression in the group (RQ ± SEM). * *p* < 0.05 (Student’s t-test).

Analysis of the expression of the *CASP3* gene revealed significantly statistically higher expression in the peripheral blood lymphocytes of patients with CD (*p* = 0.003) and patients with UC (*p* = 0.001) compared to controls. No significant differences in the level of expression of *CASP3* were shown between patients with CD and UC (*p* = 0.755) (Figure 3).

**Figure 4:**
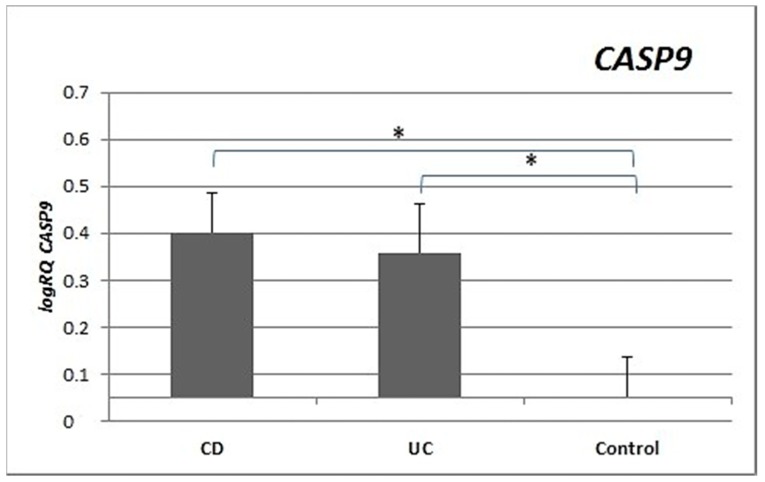
Mean expression (RQ) of the *CASP9* gene at the transcript level in peripheral blood lymphocytes of patients with UC and CD relative to the mean expression of the gene in the peripheral blood lymphocytes of the control group (calibrator). The result shows the average relative expression in the group (RQ ± SEM). * *p* < 0.05 (Student’s t-test).

Expression of the *CASP9* gene was significantly higher and more than double in the peripheral blood lymphocytes of CD patients (*p* = 0.029) and UC patients (*p* = 0.018) compared to controls. There were no statistically significant differences in *CASP9* gene expression between the CD and UC groups (*p* = 0.986) (Figure 4).

**Figure 5:**
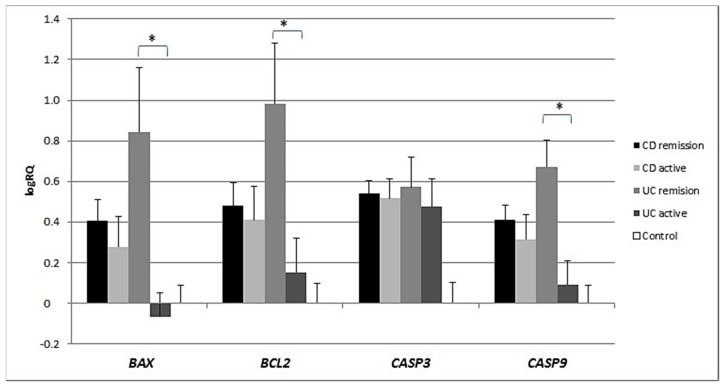
Shows the mean expression (RQ) of the *BAX*, *BCL2*, *CASP3* and *CASP9* genes at the transcript level in the peripheral blood lymphocytes of CD and UC patients during acute exacerbation with the mean expression of these genes in the peripheral blood lymphocytes during remission.

Analysis of the expression of the *BAX*, *BCL2*, *CASP3* and *CASP9* genes in patients in the active phase of their disease showed that the expression of *BAX* (*p* = 0.001), *BCL2* (*p* = 0.038) and *CASP9* (*p* = 0.007) was significantly lower in the peripheral blood lymphocytes of UC patients during acute exacerbation than in patients in remission. There were no significant differences in gene expression between CD patients during exacerbation of the disease and patients in remission. However, the expression of *BA*X (*p* = 0.047) and *BCL2* (*p* = 0.049) was significantly different between UC patients in remission compared to CD patients in remission (Figure 5).

**Figure 6:**
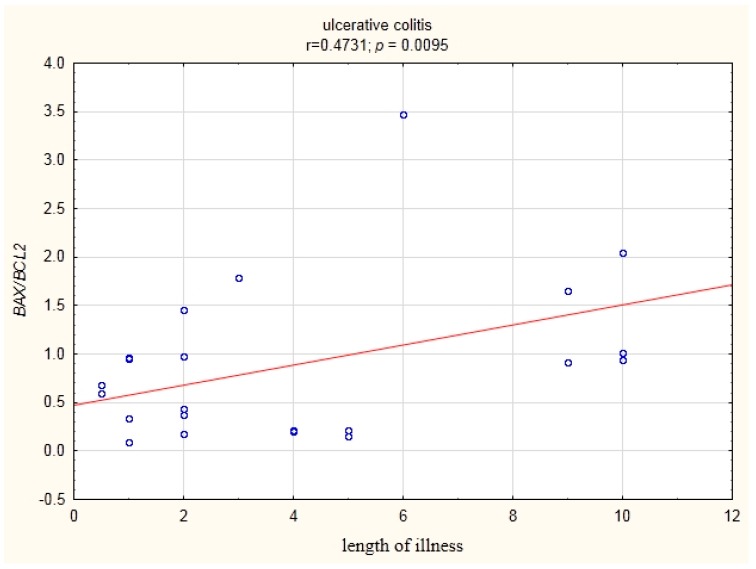
Correlation of the BAX/BCL2 ratio in the peripheral blood lymphocytes with the duration of the disease in UC patients.

In patients with UC, a statistically significant correlation (r = 0.473) of the BAX/BCL2 ratio with the duration of disease was demonstrated (Figure 6).

**Figure 7:**
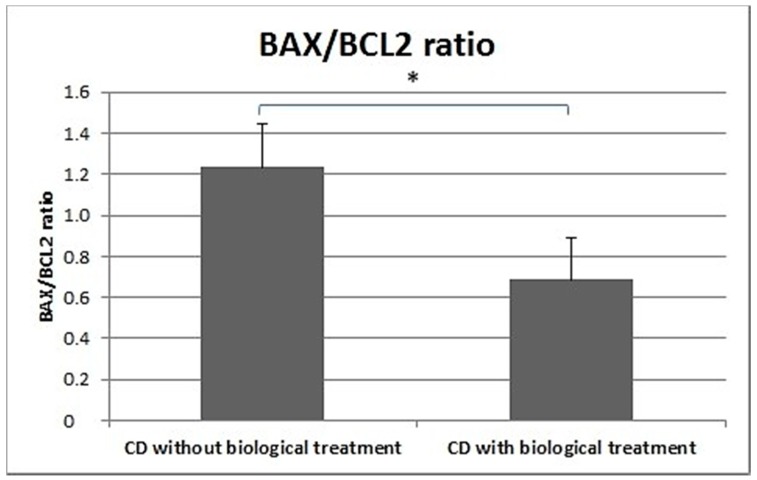
BAX/BCL2 ratio in CD patients undergoing biological treatment and CD patients not receiving biological treatment.

In the peripheral blood lymphocytes of CD patients treated biologically, a significantly lower BAX/BCL2 ratio was demonstrated compared to patients that were not biologically treated (Figure 7).

**Figure 8:**
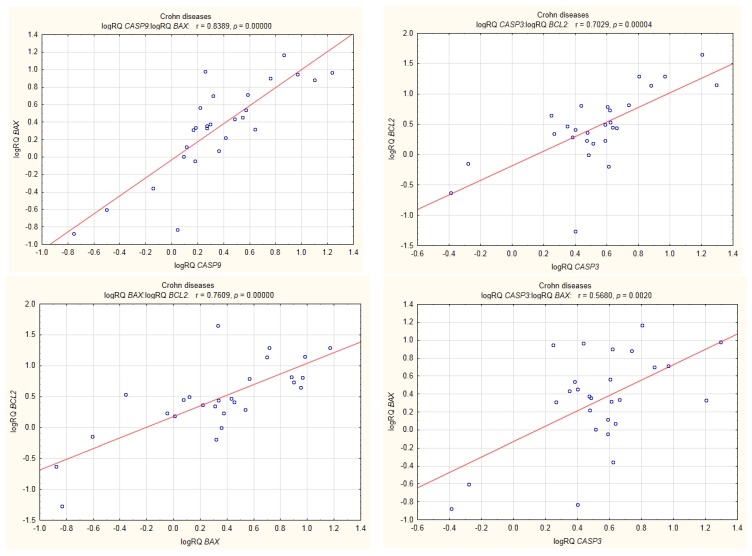
Correlations of genes in peripheral blood lymphocytes of patients with CD.

Among UC patients, *BAX* expression was shown to be positively correlated with *BCL2* expression, and the expression of *BCL2* correlated positively with that of *CASP3* (r = 0.64, *p* < 0.05). Statistically significant correlations were also shown between the expression of *BAX* and *CASP9* and between the expression of *BCL2* and *CASP9* (Figure 8).

**Figure 9:**
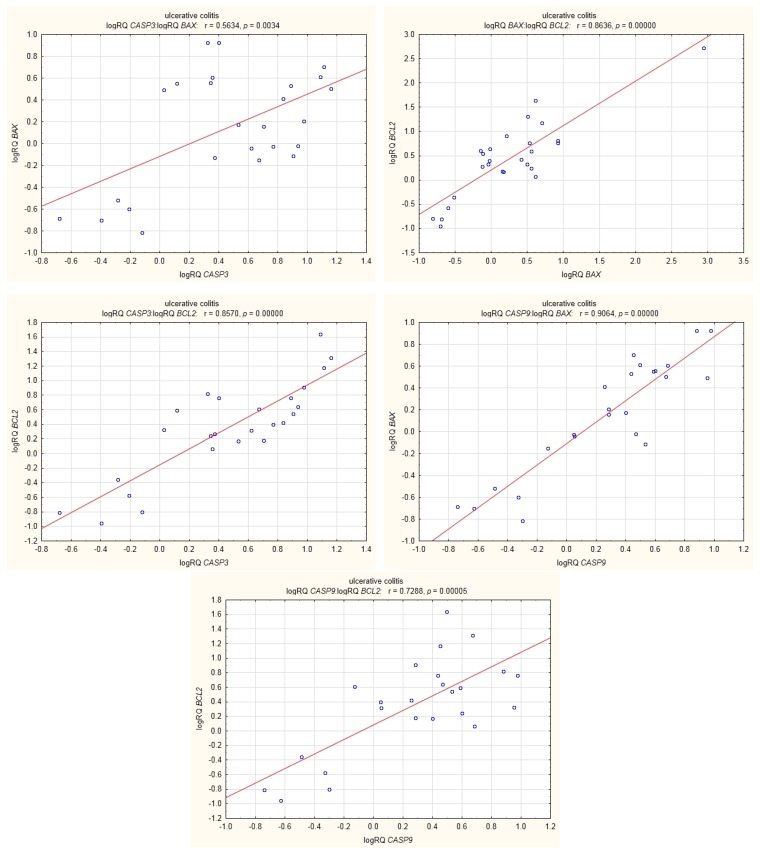
Correlations of genes in peripheral blood lymphocytes of patients with UC.

In UC patients, statistically significant positive correlations were shown for the expression of *BCL2* with *CASP3*; *CASP3* with *BAX*; *BAX* with *BCL2*; *CASP9* with *BCL2*; and *CASP9* with *BAX* (Figure 9).

An analysis was also performed of the relationship between the expression of the *BAX*, *BCL2*, *CASP3* and *CASP9* genes in CD and UC patients with parameters of blood morphology and biochemistry, i.e. leukocyte count, erythrocyte count and CRP level, as well as with treatment, age, gender and duration of the disease. In the case of age, gender, treatment, diseaseduration, CRP level and leukocyte level, no statistically significant correlations or differences in the level of gene expression were found.

## 4. Discussion

Inflammation in IBD is sustained by an exaggerated response of lymphocytes [9]. Important differences in the regulation of apoptosis between UC and CD have been identified [10]. This is supported by our research, which showed differences in the expression of apoptotic genes between CD and UC. CD patients showed statistically significantly higher expression of both the *BAX* gene (*p* = 0.012) and the *BCL2* gene (*p* = 0.022) compared to healthy controls. In contrast, in patients with ulcerative colitis, no significant differences were observed for either *BAX* (*p* = 0.32) or *BCL2* (*p* = 0.249) expression compared to controls (Figure 1 and Figure 2). The differences in gene expression in the two diseases may result from their heterogeneous nature, although both involve inflammation and ulcerations of the gastrointestinal tract [11,12].

*CASP3* expression was statistically significantly higher in the peripheral blood lymphocytes of both CD and UC patients compared to the control group. Expression of *CASP9* in the peripheral blood lymphocytes of CD (*p* = 0.029) and UC (*p* = 0.018) patients was also statistically higher (Figure 3 and Figure 4) than in healthy subjects. Our results indicate that excessive apoptosis of peripheral blood mononuclear cells occurs in patients with IBD.

Research by El-Hodhod et al. [13] has demonstrated increased apoptosis in peripheral blood lymphocytes in children with IBD. According to the authors, the increase in apoptosis of circulating lymphocytes could be a protective mechanism against organ injury, as increased lymphocyte apoptosis can be linked to the secretion of anti-inflammatory cytokines and may thereby prevent an unwanted immune response and organ injury. Increased apoptosis of circulating lymphocytes may also be attributed to nutrient deficiency, which is very common in IBD patients.

In the group of UC patients, we found a strong statistically significant positive correlation between the level of expression of *BCL2* and *CASP3*. In the group of CD patients, the expression of *BCL2* was also positively correlated with that of *CASP3*, but to a lesser extent (Figure 8 and Figure 9). This may explain the fact that in UC the cell’s defence mechanisms protect it against apoptosis to a greater degree by increasing anti-apoptotic *BCL2*. These results may be supported by research carried out by Sventoraityte et al. [14], which demonstrated that in IBD patients the imbalance between production of pro-inflammatory Th1 and anti-inflammatory Th2 cytokines persists even during remission of the disease, and disturbances of immune homeostasis are significantly more expressed in patients with CD than in patients with UC.

In our research, among the group of UC patients, the expression of *BAX*, *BCL2* and *CASP9* was significantly statistically lower in the subjects during relapse than in patients in remission, while in CD no significant changes in gene expression depending on disease exacerbation were observed (Figure 5). In a review paper, Neuman [15] also points out that *BAX* expression was significantly lower in UC patients during inflammation. Goretsky et al. [16] emphasize that the acute phase of IBD is characterized by the overproduction of tumour necrosis factor (TNF) in the intestinal mucosa. For this reason, treatment of IBD is often directed against tumour necrosis factor-α (TNF-α), and the induction of apoptosis in activated monocytes or T lymphocytes may be treated as a therapeutic tool in IBD.

A number of studies suggest varying efficacy of treatments using anti-TNF-α antibodies in UC patients, while it is more successful in CD patients. In a study by Trinder and Lawrance [17], treatment with Adalimumab proved more effective in the case of patients with CD than in UC patients [17], which is indicative of differences in the two diseases [11,12] and in the apoptosis pathways in them. This is supported by our study, which showed significant differences in the expression of apoptosis genes *BAX* and *BCL2* in CD and UC in remission, with significantly higher expression of these two genes in UC compared to CD in remission.

No significant differences in the expression of *BAX*, *BCL2*, *CASP3* or CASP9 were shown between subjects suffering from CD and undergoing biological treatment (Humira or Inflectra), and CD patients who were not receiving such treatment. However, *CASP3* expression was significantly higher both in subjects undergoing biological treatment and those who were not in comparison to the control group, which may suggest increased apoptosis of T lymphocytes in both groups.

Research by Schmitz et al. [18] demonstrated the clinical effectiveness of biological treatment [18], while Blander [19] noted that there is a group of IBD patients who are not responsive to anti-TNF-α treatment. These patients display a slight change or none at all in clinical symptoms and a lack of macroscopicmucosalhealing following anti-TNF-α therapy.

Several studies have demonstrated that exaggerated activation of lymphocytes contributes to the pathogenesis of IBD, and therefore, medical therapies are linked to BCL2-family-mediated apoptosis. An imbalance in BCL2 family proteins may cause failure in therapeutic responses [2].

Our study showed a positive statistically significant correlation (r = 0.473) between the BAX/BCL2 ratio and disease duration in the group of UC patients (Figure 6).

Furthermore, the BAX/BCL2 ratio was significantly lower in the peripheral blood leukocytes of CD patients receiving biological treatment (Humira or Inflectra) than in CD patients not receiving such treatment (Figure 7).

Our results may suggest that an underlying imbalance in factors controlling apoptosis in peripheral blood mononuclear cells may be the response of the immune system to inflammation in the intestinal mucosa. We may also hypothesize that an underlying imbalance in factors controlling apoptosis in peripheral blood mononuclear cells may after some time lead to inflammatory bowel disease (by maintaining chronic inflammation).

Some studies have shown that the modulation of apoptosis in peripheral blood lymphocytes and the colonic mucosa may help alleviate inflammation [2].

Lutz et al. [2] have investigated the role of BCL2 inhibitor ABT-737 in lymphocyte apoptosis in mice under inflammatory conditions. Apoptosis was found to increase following treatment with ABT-737 in the lymphocytes, splenocytes and Peyer’s patches. The inhibitor positively altered the colonic mucosa and ameliorated inflammation, as shown by colonoscopy, histology and colon length. The BIM/BCL2 ratio decreased with age and during the course of treatment, and long-term treatment resulted in adapted TNF levels.

This study and our results suggest that the modulation of apoptosis may affect the course of IBD.

Our study has shown significant expression of the genes *BAX* and *BCL2* in CD, which indicates dysregulation of apoptosis and suggests that modulation of apoptosis may become an important therapeutic mechanism in IBD.

Due to the fact that the small size of the sample may limit the interpretation of our findings, it is necessary to carry out large studies aiming to clarify this matter.

## 5. Conclusions

Our research has shown a simultaneous increase in the expression of the anti-apoptotic gene *BCL2* and the proapoptotic gene *BAX*, which suggests the dysregulation of apoptosis mechanisms in IBD.

The significantly higher expression of genes *BAX* and *BCL-2* in UC and CD patients in remission may suggest differences in these diseases in terms of their prognosis and treatment.

Our results may suggest that an underlying imbalance in factors controlling apoptosis in peripheral blood lymphocytes may be the response of the immune system to inflammation of the intestinal mucosa.

Modulation of apoptosis may become an important therapeutic mechanism in IBD.

## Figures and Tables

**Table 1:** Demographic and clinical characteristics of CD and UC patients and control subjects.

Variable	CTR, *n* = 35	CD, *n* = 27	UC, *n* = 28
**Age (Years)**	40 ± 9.11	34.7 ± 11.1	37.7 ± 15.11
**Gender (M/F)**	9 M, 26 F	14 M, 13 F	12 M, 16 F
**Disease Duration (Years)**		6.68 ± 4.8	4.96 ± 4.69
**Disease Phase (Active/Remission)**		19 active, 8 remission	19 active, 9 remission
**Inflammation localized**		ileocaecalregion, caecum and bauhin’svalve, ileum	pancolitis, rectum, sigmoid colon, transverse colon, sigmoid and rectum

CD-Crohn’s disease, CTR-control group, UC-ulcerative colitis.
